# Description of three new species of *Aposphragisma* Thoma, 2014 (Araneae: Oonopidae) from Sumatra, Indonesia

**DOI:** 10.3897/zookeys.797.29364

**Published:** 2018-11-19

**Authors:** Riko Fardiansah, Nadine Dupérré, Rahayu Widyastuti, Anton Potapov, Stefan Scheu, Danilo Harms

**Affiliations:** 1 Zoological Museum, Center of Natural History, Universität Hamburg, Martin-Luther-King-Platz 3, 20146 Hamburg, Germany; 2 Institut Pertanian Bogor – IPB, Department of Soil Sciences and Land Resources, Dramaga Campus, Bogor 16680, Indonesia; 3 University of Göttingen, J.F. Blumenbach Institute of Zoology and Anthropology, Untere Karspüle 2, 37073 Göttingen, Germany; 4 University of Göttingen, Centre of Biodiversity and Sustainable Land Use, Von-Siebold-Str. 8, 37075 Göttingen, Germany

**Keywords:** Arachnida, biodiversity, goblin spiders, systematics, taxonomy

## Abstract

Three species from the family Oonopidae are newly described from leaf litter habitats in Sumatra, Indonesia based on male and female morphology. All three species belong to the genus *Aposphragisma* Thoma, 2014: *Aposphragismaglobosum***sp. n.**, *Aposphragismajambi***sp. n.**, and *Aposphragismasumatra***sp. n.**

## Introduction

The family Oonopidae Simon, 1890 is a diverse group of spiders with over 1801 described species in 114 genera from all over the world (World Spider Catalog 2018). Oonopids are small (1–4 mm), two-clawed, ecribellate spiders (Saaristo 2001, Jocqué and Dippenaar-Schoeman 2007) that can be abundant in leaf litter, under bark of trees, in forest canopies and in subterranean habitats (Burger et al. 2002; Harvey and Edward 2007; Fannes and Jocqué 2008; Baehr et al. 2010). Currently 42 species of Oonopidae belonging to eight genera are known to occur in Indonesia, excluding Borneo (World Spider Catalog 2018). More than half of the species can be found on Sumatra (23 species), as well as most of the genera (six genera). The most species-rich genus, *Ischnothyreus* Simon, 1893 was recently revised by Richard et al. (2016) with eight species occurring on the island. It is followed by the genera *Gamasomorpha* Karsch, 1881 and *Prethopalpus*Baehr et al. 2012 (with six species each) revised by Eichenberger et al. (2011) and Baehr et al. (2012), respectively.

In 2014, Thoma described the new South East Asian genus *Aposphragisma* Thoma, 2014 including 19 new species, from which only one species was described from Sumatra, *A.borgulai* Thoma, 2014 (Thoma et al. 2014). Here we present the description of three new species of *Aposphragisma* from Sumatra.

## Material and methods

All specimens were collected in the framework of the EFForTS (Ecological and Socio-economic Functions of Tropical Lowland Rainforest Transformation Systems) project that investigates the effects of transformation of lowland rainforests into agricultural systems (Drescher et al. 2016). Samples were taken from four forest types (primary degraded lowland rainforest, agroforest with a mixture of native vegetation and planted rubber trees (secondary degraded lowland rainforest), rubber monoculture, and oil palm monoculture). Material was collected during three sampling campaigns. The first was conducted in October–November 2012 (Barnes et al. 2014). From each of 32 sampling sites, three samples of 1×1 m were taken by sieving the leaf litter through a sieve of 2-cm mesh. Spiders visible by eye were hand-collected and stored in 65% ethanol. The second campaign was conducted in October–November 2013 (Klarner et al. 2017). From the same sampling sites three soil samples of 16×16 cm were taken with a spade, comprising the litter layer and the underlying mineral soil to a depth of 5 cm. Finally, the third collecting round was conducted in March, June, September and December 2017; specimens were collected by sieving 16×16cm of litter. All spiders from soil and litter were extracted by heat (Kempson et al. 1963) and collected in a dimethyleneglycol-water solution (1 : 1) and thereafter transferred into 70% ethanol. The material examined is deposited in the following institutions: Indonesian Institute of Sciences Cibinong, Indonesia (**LIPI**); Zoological Museum Hamburg, Germany (**ZMH**).

Specimens were examined in 65–75% ethanol under a Leica M125 dissection microscope and photographed with a custom-made BK Plus Lab System by company Dun, Inc. with integrated Canon camera, macro lenses (65 mm and 100 mm) and the Zerene stacking software (Zerene Systems LLC 2018). Female genitalia were excised using a sharp entomological needle, treated with Pancreatin (Álvarez-Padilla and Hormiga 2008), then placed in lactic acid and observed under a Leica DM2500 LED compound microscope. A Leica DMC 4500 digital camera attached to the microscope was used to photograph all the structures illustrated. All measurements are given in millimetres (mm). Males and females were matched based on the following criteria: (1) collected in the same sample, (2) body size, and (3) punctuation pattern and colouration. Morphological nomenclature follows Thoma et al. (2014).

**Abbreviations**

**Somatic morphology**:

ALE anterior lateral eyes;

bc book lung cover;

d denticles;

lap lateral apodemes;

PME posterior median eyes;

PLE posterior lateral eyes;

sli slit;

s spikes;

sr subterminal scutal ridges;

st sternum tubercle;

tlp tooth-like projection;

**Male genitalia**:

cb conical bulge;

c conductor;

e embolus;

sp sperm pore;

**Female genitalia**:

gap globular appendix;

na nail-like structure;

pa papillae;

re receptaculum;

sa sac-like structure;

tsc transverse sclerites;

wl wrinkle-lines;

## Taxonomy

### Family Oonopidae Simon, 1890

#### Genus Aposphragisma

Taxon classificationAnimaliaAraneaeOonopidae

Thoma, 2014

##### Type species.

*Aposphragismahelvetiorum* Thoma, 2014: 36–44

##### Diagnosis.

The genus *Aposphragisma* most resembles the genera *Gamasomorpha* Karsch, 1881 and *Xestaspis* Simon, 1884 (Thoma et al. 2014) but they can be differentiated based on the combination of the following characters: hard-bodied spiders; sternum with microsculpture; dorsal and ventral abdominal scuta not fused; labium strongly incised; legs without spines. Furthermore, males are distinguished by their palpal bulb bearing an apical embolus closely associated with a laminar conductor (Thoma et al. 2014).

##### Distribution.

Borneo, Indonesia (Sumatra), Malaysia, Singapore and Vietnam.

#### Aposphragisma globosum

Taxon classificationAnimaliaAraneaeOonopidae

Fardiansah & Dupérré, sp. n.

http://zoobank.org/30F29C2E-E74A-4847-BAE1-1D83A77C2AAD

Figs 1 , 2 , 3

##### Type material.

**Holotype**. ♂: Indonesia, Sumatra, Harapan, 02°09'09.9"S 103°21'43.2"E, secondary lowland rainforest, 26 November 2017, B. Klarner (LIPI). **Paratypes.** 1♀, Indonesia, Sumatra, Harapan, 01°54'35.6"S 103°15'58.3"E, oil palm plantation, October 2012, M. Jochum, A. Barnes (LIPI); 01°54'39.5"S 103°16'00.1"E, 2♂, rubber plantation, October 2013, B. Klarner (ZMH–A0000984, ZMH–A0000986); 02°09'09.9"S 103°21'43.2"E, 1♂2♀, secondary lowland rainforest litter, October 2013, B. Klarner (ZMH–A00001002, ZMH–A0001022, ZMH–A0001505); 01°55'44.0"S 103°15'33.8"E, 2♀, agroforest with a mixture of native vegetation and planted rubber trees, October 2012, M. Jochum, A. Barnes (ZMH–A0001304, ZMH–A0001305).

##### Etymology.

The specific name is an adjective in apposition taken from Latin, meaning *globular* in reference to the shape of female genitalia.

##### Diagnosis.

**Figure 1.**
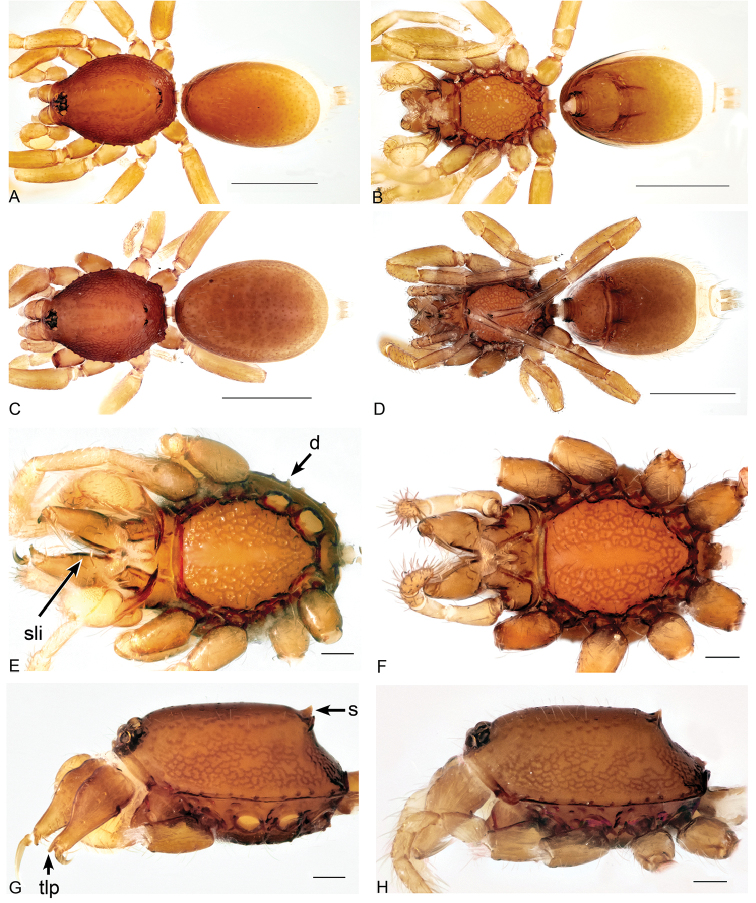
*Aposphragismaglobosum* sp. n., Male (**A, B, E, G**); Female (**C, D, F, H**). **A, C** habitus dorsal view **B, D** habitus ventral view **E, F** prosoma ventral view **G, H** carapace lateral view. Scale bars: 0.5mm (**A–D**); 0.1mm (**E–H**).

**Figure 2.**
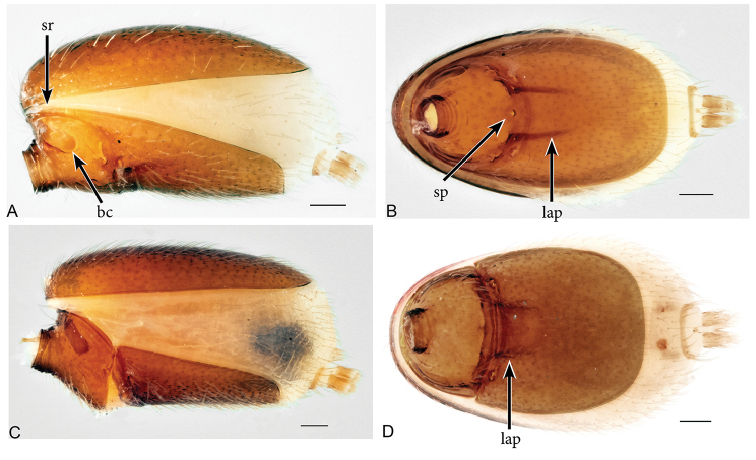
*Aposphragismaglobosum* sp. n., Male (**A, B**); Female (**C, D**). **A, C** abdomen lateral view **B, D** abdomen ventral view. Scale bar: 0.1mm (**A–D**).

**Figure 3.**
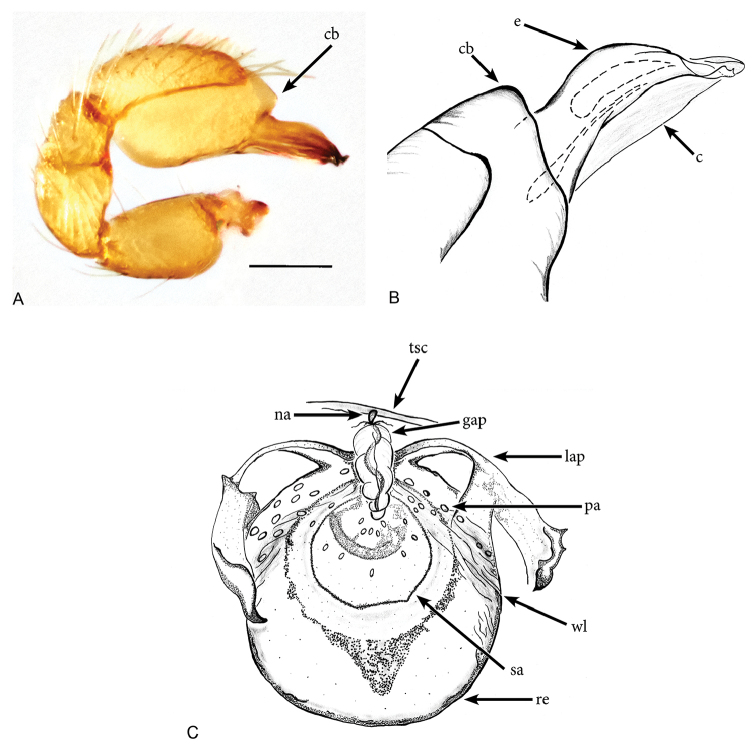
*Aposphragismaglobosum* sp. n., Male (**A, B**); Female (**C**). **A, B** palp prolateral view **C** female genitalia dorsal view Scale bar: 0.1mm (**A**).

*Aposphragismaglobosum* sp. n. males and females can be distinguished from most *Aposphragisma* species by the presence of prosomal spikes (Fig. 1G) and a coarsely reticulated sternum (Fig. 1F). From *A.brunomanseri* Thoma, 2014 it can be separated by the presence of only one pair of prosomal spikes (Fig. 1G), the latter species having two pairs of spikes (Thoma et al. 2014; fig. 2E, F); males are differentiated from *A.kolleri* Thoma, 2014 by their strongly twisted tip of the embolus (Fig. 3B), which is spatulate in the latter species (Thoma et al. 2014; fig. 27C).

##### Description.

***Male (holotype)*.** Total length: 1.54; carapace length: 0.69; carapace width: 0.53; abdomen length: 0.85; abdomen width: 0.51. Cephalothorax. *Carapace*: Brownish orange, broadly oval in dorsal view, slightly elevated in lateral view, surface of elevated portion of pars cephalica smooth and with 1 pair of spikes at the apical end; sides and pars thoracica finely reticulated, partly interrupted by small smooth areas, lateral margin with blunt denticles (Fig. 1A, G). *Sternum*: Brownish orange; longer than wide, coarsely reticulated except of median stripe and smooth edges, covered by sparse setae; posterior margin extending posteriorly (Fig. 1E). *Eyes*: Six; ALE largest, ALE oval, PME circular, PLE oval; posterior eye row straight from above, procurved from front; ALE separated by their diameter, ALE-PLE separated by less than ALE radius, PMEs joint together, PLE-PME separated by less than PME radius (Fig. 1A). *Mouthparts*: chelicerae yellowish brown; straight in frontal view, convex in lateral view; anterior face strongly indented; posterior margin of inner surface proximally modified to a ridge with median slit (sli); promargin with row of flattened setae that extend distally into a short inward-pointing tooth-like projection (tlp) (Fig. 1G). Labium triangular, deeply incised, fused to sternum (Fig. 1E). Endites elongated; outer margin subdistally with a pair of long inward-bent setae. Abdomen. Dorsal scutum brownish yellow, ovoid; strongly sclerotized, covering most of dorsum (Fig. 1A); epigastric scutum strongly sclerotized, anteriorly with subterminal scutal ridge (sr) which is widely oval (Fig. 2A); book lung covers large (bc), oval (Fig. 2A). Postepigastric scutum long, strongly sclerotized, occupying most of the venter, with posteriorly directed lateral apodemes (lap) (Fig. 2B). *Legs*: yellowish orange. Genitalia. *Epigastric region*: sperm pore (sp) situated at level of posterior spiracles (Fig. 2B). *Palp*: Yellowish bright, not strongly sclerotized (Fig. 3A); cymbium slightly ovoid in dorsal view; bulb stout; conical bulge (cb) strongly pronounced (Fig. 3A, B); embolus (e) tip twisted; conductor (c) with blunt tip (Fig. 3B).

***Female (paratype)*.** Total length: 1.75; carapace length: 0.77; carapace width: 0.55; abdomen length: 0.98; abdomen width: 0.65. Colouration: same as in male. Cephalothorax. *Carapace*: Same as in male. *Mouthparts*: chelicerae distally without pointing tooth-like projection. Abdomen. Epigastric scutum not fused to postepigastric scutum (Fig. 2D); postepigastric scutum with posteriorly directed lateral apodemes (lap) (Fig. 2D). *Legs*: yellowish white. Genitalia. Dorsal view (Fig. 3C): receptaculum (re) large, globular, convex and sloping upward containing a globular sac-like structure (sa); anterior part covered with papillae (pa), mediolateral with wrinkle-like lines (wl); laterally framed by rectangular sclerites (apodemes, lap) that have wide and slightly folded tips, apodemes sloping upward; globular appendix (gap) lying dorsally of receptaculum and about 1/3 as long as receptaculum; a transverse sclerite (tsc) lies anteriorly to the receptaculum and bears a nail-like structure (na) more medially.

##### Natural History.

Specimens were collected in four types of habitats: secondary lowland rainforest, oil palm plantation, and rubber plantation.

##### Distribution.

Known only from the type locality: Harapan on Sumatra.

#### Aposphragisma jambi

Taxon classificationAnimaliaAraneaeOonopidae

Fardiansah & Dupérré, sp. n.

http://zoobank.org/C8D21542-5234-48E1-BCC0-9AA0314AB504

Figs 4 , 5 , 6

##### Type material.

**Holotype** ♂: Indonesia, Sumatra, Bukit Duabelas, 01°59'42.5"S 102°45'08.1"E, secondary lowland rainforest, October 2012, M. Jochum, A. Barnes (LIPI). **Paratypes**: 1♀, Indonesia, Sumatra, Bukit Duabelas, 02°08'26.6"S 102°51'04.3"E, agroforest with a mixture of native vegetation and planted rubber trees, October 2012, M. Jochum. A. Barnes (LIPI); 01°59'42.5"S 102°45'08.1"E, 2♀, secondary lowland rainforest litter, October 2013, B. Klarner (ZMH–A0000994, ZMH–A0000998), 01°59'42.5"S 102°45'08.1"E, 2♀, October 2012, M. Jochum, A. Barnes (ZMH–A0001273, ZMH–A0001282).

##### Etymology.

The specific name is a noun in apposition and refers to the name of Jambi Province where Bukit Duabelas National Park is located.

##### Diagnosis.

**Figure 4.**
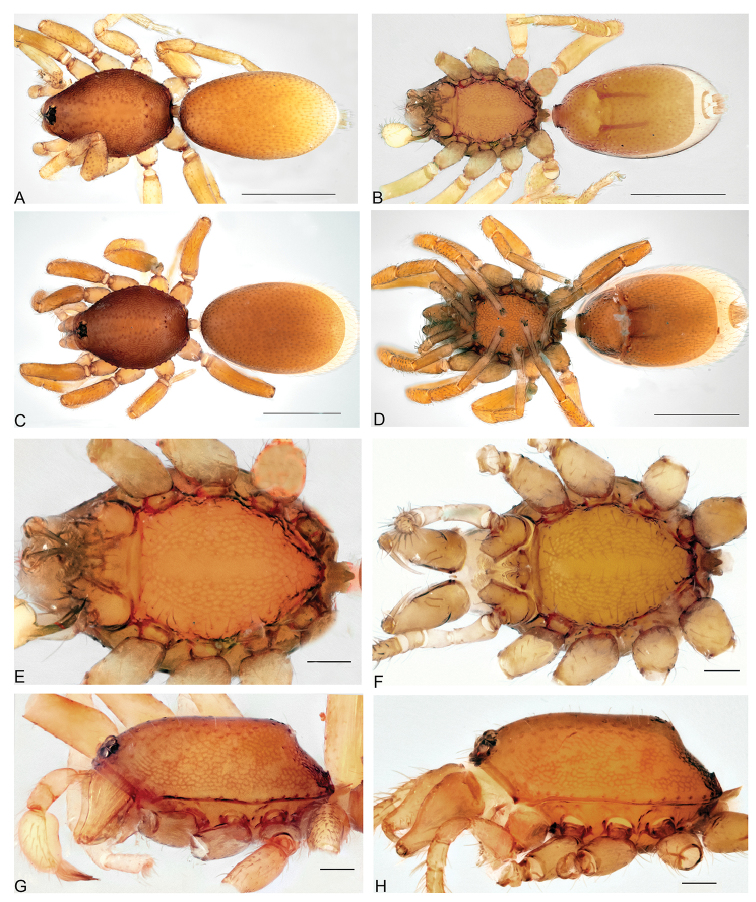
*Aposphragismajambi* sp. n., Male (**A, B, E, G**); Female (**C, D, F, H**). **A, C** habitus dorsal view **B, D** habitus ventral view **E, F** prosoma ventral view **G, H** carapace lateral view. Scale bars: 0.5mm (**A–D**); 0.1mm (**E–H**).

*Aposphragismajambi* sp. n. males and females can be distinguished from most of the other *Aposphragisma* species by their finely reticulate carapace lacking spikes (Fig. 4A, C, G, H) and by their finely reticulated sternum with smooth median stripe (Fig. 4E, F); from *A.baltenspergerae* Thoma, 2014 and *A.retifer* Thoma, 2014 males are differentiated by their wider and strongly twisted embolus tip (Fig. 6B), not twisted in *A.baltenspergerae* and *A.retifer* (Thoma et al. 2014; figs. 4C and 37E respectively) and females by their elongated oval receptaculum (Fig. 6C), globose in *A.baltenspergerae* (Thoma et al. 2014; fig. 4G).

##### Description.

**Figure 5.**
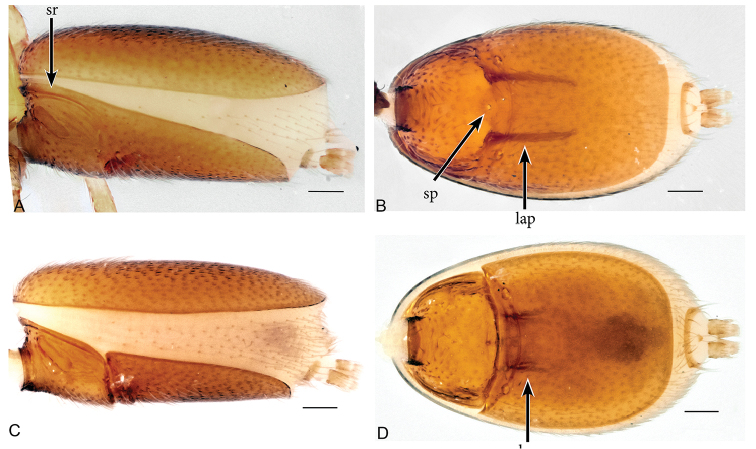
*Aposphragismajambi* sp. n., Male (**A, B**); Female (**C, D**). **A, C** abdomen lateral view **B, D** abdomen ventral view. Scale bar: 0.1mm (**A–D**).

**Figure 6.**
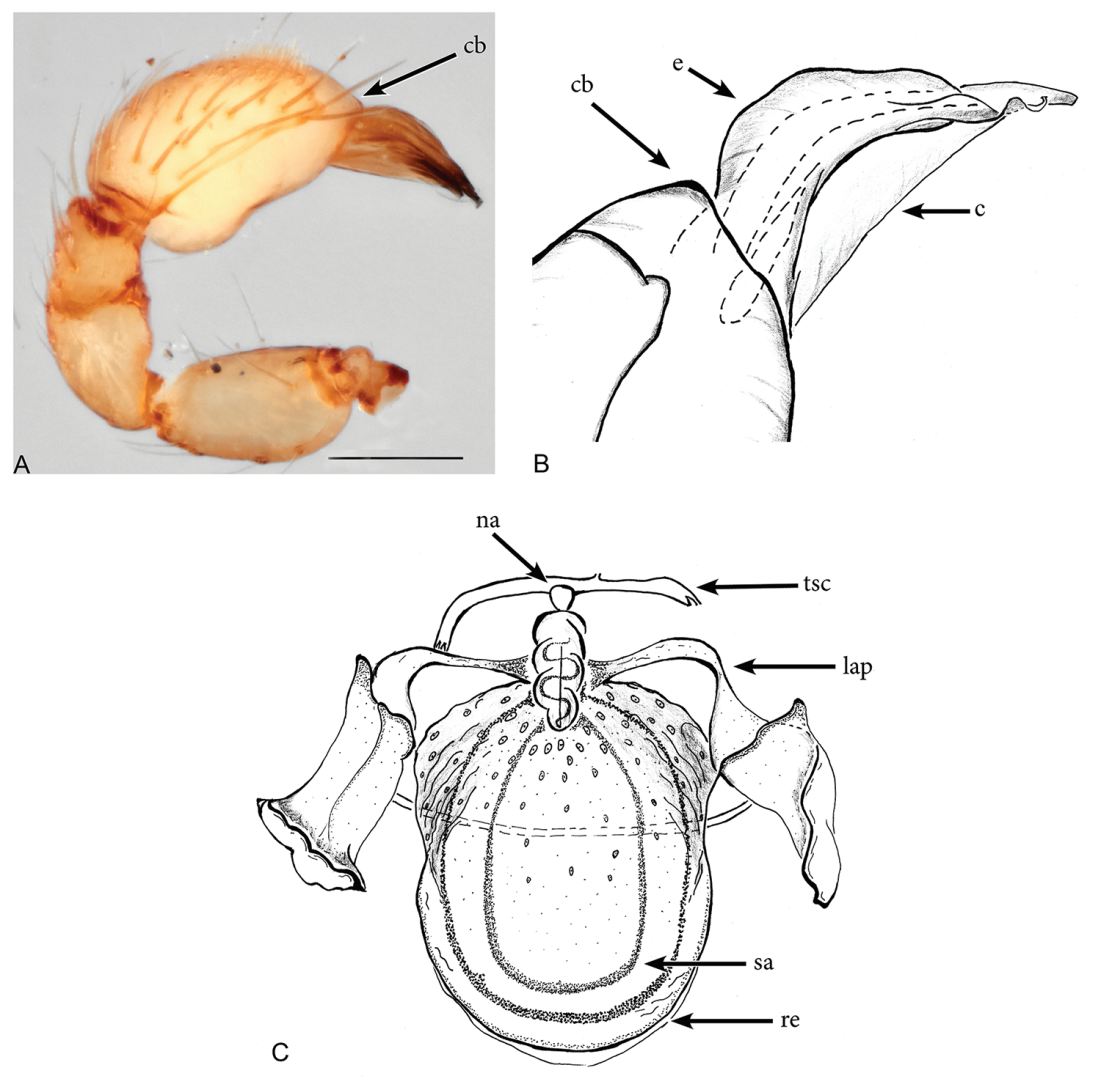
*Aposphragismajambi* sp. n., Male (**A, B**); Female (**C**). **A, B** palp prolateral view **C** female genitalia dorsal view. Scale bar: 0.1mm (**A**).

***Male (holotype).*** Total length: 1.54; carapace length: 0.68; carapace width: 0.47; abdomen length: 0.86; abdomen width: 0.48. Cephalothorax. *Carapace*: Brownish orange, broadly oval in dorsal view, slightly elevated in lateral view, surface of elevated portion of pars cephalica smooth and without spikes, with 2 small tubercles at apical end that each bears a seta (Fig. 4A, G); sides of carapace finely reticulated; pars thoracica finely reticulated, sloping gradually; lateral margin with blunt denticles (Fig. 4G). *Sternum*: Brownish orange; longer than wide, finely reticulated except median stripe and broadly smooth edges, surface covered by setae (Fig. 4E). *Eyes*: Six, all oval; ALE largest, posterior eye row straight from above; ALE separated by less than its radius, ALE-PLE touching, PME touching, PLE-PME touching (Fig. 4A). *Mouthparts*: chelicerae yellowish white, slightly divergent, anterior face convex in lateral view (Fig. 4G), inner surface covered by thick setae; posterior margin of inner surface proximally modified into a ridge with median slit; promargin with a row of flattened setae, distally extending into a short inwards-pointing tooth-like projection. Labium triangular, deeply incised, fused to sternum (Fig. 4E). Endites elongated, outer margin subdistally with a pair of long inward-bent setae. Abdomen. Dorsal scutum brownish yellow, ovoid; strongly sclerotized and covering full length of abdomen (Fig. 4A); book lung covers large, ovoid, surface smooth (Fig. 5A); epigastric scutum strongly sclerotized, anteriorly with widely oval subterminal, scutal ridge (sr) (Fig. 5A). Postepigastric scutum long, strongly sclerotized and occupying most of the venter, posteriorly-directed long lateral apodemes (lap) (Figs 4B, 5B). *Legs*: yellowish white. Genitalia. *Epigastric region*: sperm pore (sp) situated at level of posterior spiracles (Fig. 5B). *Palp*: Light yellow, not strongly sclerotized (Fig. 6A); cymbium slightly ovoid or rectangular in dorsal view; bulb stout; conical bulge (cb) only slightly pronounced (Fig. 6A, B); embolus (e) tip folded, conductor (c) with pointed tip (Fig. 6B).

***Female (paratype)*.** Total length: 1.54; carapace length: 0.70; carapace width: 0.49; abdomen length: 0.84; abdomen width: 0.53. Colouration: Same as in male. Cephalothorax. *Carapace*: Same as in male. *Mouthparts*: Chelicerae: distally without pointing tooth-like projection. Abdomen. Dorsal scutum not covering full length of the abdomen, soft tissue visible in dorsal view (Fig. 4C); epigastric scutum not fused to postepigastric scutum (Fig. 5C, D); postepigastric scutum with short posteriorly directed lateral apodemes (lap) (Fig. 5D). *Legs*: yellowish white. Genitalia. Dorsal view (Fig. 6C): Receptaculum (re) large, elongated oval, convex and sloping upward, containing an oval sac-like structure (sa), anterior and median part covered with papillae, anterolateral with wrinkle-like lines; laterally framed by rectangular sclerites (apodemes, lap) and with a wide and slightly folded tip; apodemes sloping upward; globular appendix lying dorsally of receptaculum and about 1/4 as long as receptaculum; a transverse sclerite (tsc) lies anteriorly to the receptaculum and has a nail-like structure (na) in medial position.

##### Natural History.

Specimens were collected in a secondary degraded lowland rainforest only.

##### Distribution.

Known only from the type locality, Bukit Duabelas National Park, Sumatra.

#### Aposphragisma sumatra

Taxon classificationAnimaliaAraneaeOonopidae

Fardiansah & Dupérré, sp. n.

http://zoobank.org/97D45CA3-46CD-47A7-ADAA-9887F86711DC

Figs 7 , 8 , 9

##### Type material.

**Holotype** ♂: Indonesia, Sumatra, Harapan, 02°09'09.9"S 103°21'43.2"E, secondary lowland rainforest, 10 June 2017, B. Klarner (LIPI). **Paratypes**: 2♀, Indonesia, Sumatra, Harapan, 02°09'09.9"S 103°21'43.2"E, secondary lowland rainforest, 4 September 2017 (LIPI) (ZMH–A0001198, ZMH–A0001203); 02°09'09.9"S 103°21'43.2"E, 1♂3♀, 8 March 2017, B. Klarner (ZMH–A0001196, ZMH–A0001197, ZMH–A0001199, ZMH–A0001202), 3♂4♀, 10 June 2017, B. Klarner (ZMH–A0001195, ZMH–A0001200, ZMH–A0001204), 1♂, 26 November 2017, B. Klarner (ZMH–A0001194).

##### Etymology.

The specific name is a noun in apposition, the name of the island on which the types were collected.

##### Diagnosis.

**Figure 7.**
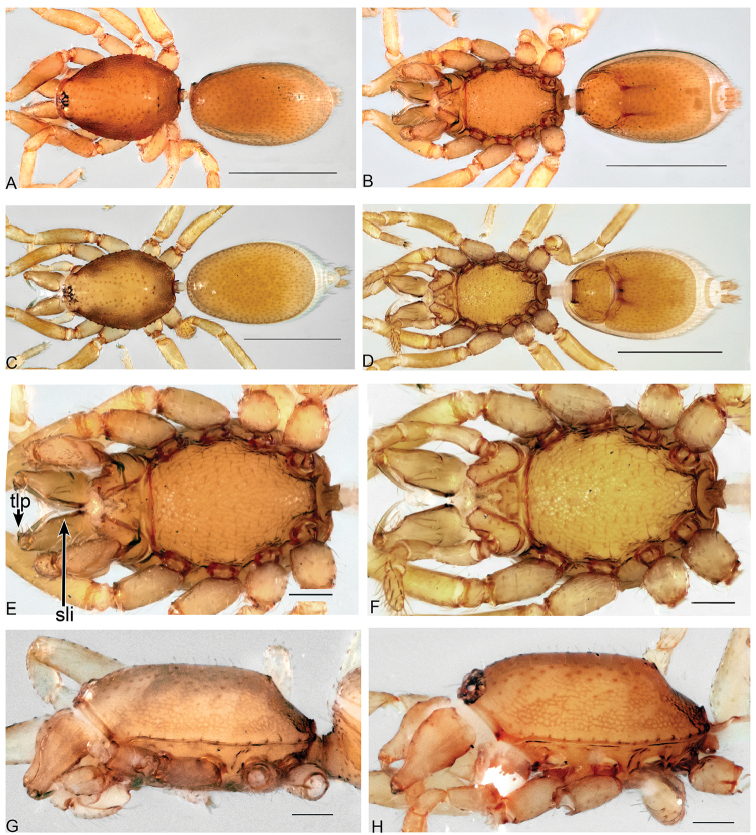
*Aposphragismasumatra* sp. n., Male (**A, B, E, G**); Female (**C, D, F, H**). **A, C** habitus dorsal view **B, D** habitus ventral view **E, F** prosoma ventral view **G, H** carapace lateral view. Scale bars: 0.5mm (**A–D**); 0.1mm (**E–H**).

*Aposphragismasumatra* sp. n. males and females can be distinguished from most of the other *Aposphragisma* species by their completely reticulate sternum (Fig. 7E, F); from *A.confluens* Thoma, 2014, *A.draconigenum* Thoma, 2014, *A.nocturnum* Thoma, 2014 and *A.scimitar* Thoma, 2014 by their blunt tubercles on the carapace margin (Fig. 7G, H); absent or reduced in the other species; from *A.stannum* Thoma, 2014 by their longer embolus (Fig. 9B), shorter in the latter species (Thoma et al. 2014; fig. 48D). From *A.rimba* Thoma, 2014, both males and females are differentiated by their reticulated carapace (Fig. 7G, H) and reduced eyes; in *A.rimba* the carapace and the eyes are of normal size (Thoma et al. 2014; fig. 38A).

##### Description.

**Figure 8.**
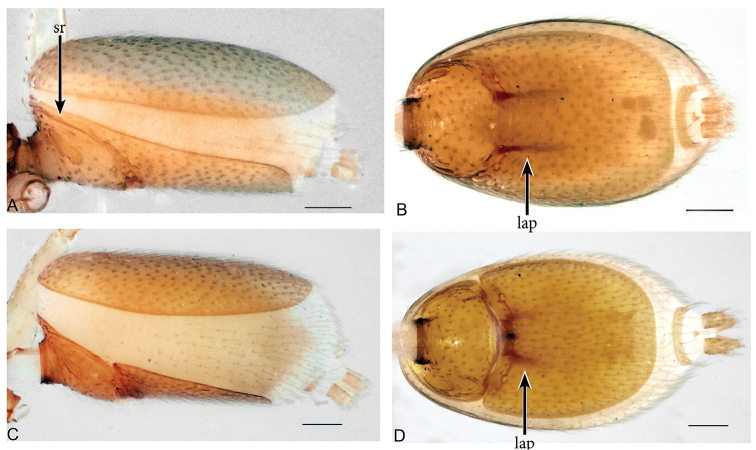
*Aposphragismasumatra* sp. n., Male (**A, B**); Female (**C, D**). **A, C** abdomen lateral view **B, D** abdomen ventral view. Scale bar: 0.1mm (**A–D**).

**Figure 9.**
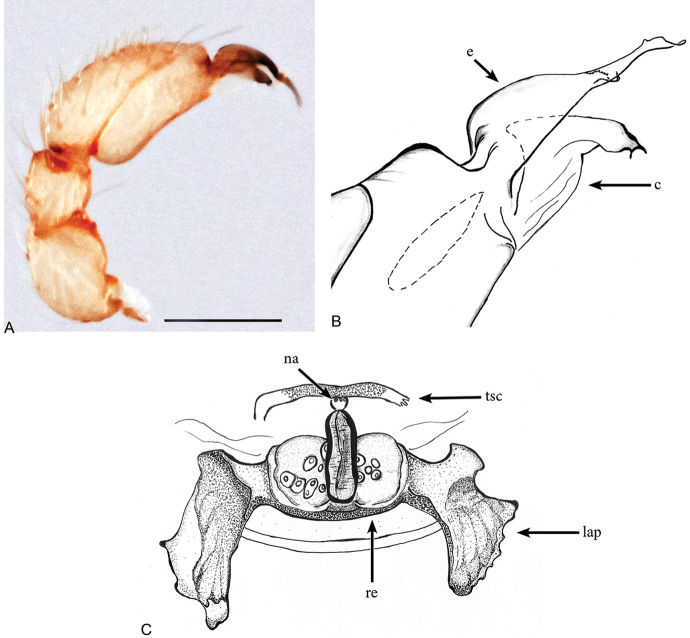
*Aposphragismasumatra* sp. n., Male (**A, B**); Female (**C**). **A, B** palp prolateral view **C** female genitalia dorsal view. Scale bar: 0.1mm (**A**).

***Male (holotype)*.** Total length: 1.28; carapace length: 0.58; carapace width: 0.41; abdomen length: 0.70; abdomen width: 0.42. Cephalothorax. *Carapace*: Brownish orange, broadly oval in dorsal view, slightly elevated in lateral view, surface of elevated portion of pars cephalica smooth and without spikes, with 2 small tubercles at apical end that bear a terminal seta (Fig. 7A, G); sides finely reticulated; pars thoracica finely reticulated, sloping gradually, lateral margin with blunt denticles (Fig. 7G). *Sternum*: Brownish orange; longer than wide, finely reticulated, surface covered with setae (Fig. 7E). *Eyes*: Six, reduced, all oval; ALE largest; posterior eye row straight from above; ALE separated by their diameter, ALE-PLE separated by less ALE radius, PME separated by less than its radius, PLE-PME separated by less than its radius (Fig. 7A). *Mouthparts*: chelicerae yellowish white, straight in frontal view, convex in lateral view (Fig. 7G); posterior margin of inner surface proximally modified to a ridge with a median slit (sli); promargin with a row of flattened setae, distally extending into a short inward-pointing tooth-like projection (tlp) (Fig. 7E). Labium triangular, deeply incised, fused to sternum (Fig. 7E). Endites elongated, outer margin subdistally with a pair of long inward bent setae. Abdomen. Dorsal scutum yellowish white, ovoid; strongly sclerotized and covering full length abdomen (Fig. 7A); epigastric scutum strongly sclerotized, anteriorly with widely oval subterminal scutal ridge (sr) (Fig. 8A), book lung covers large, ovoid, surface smooth (Fig. 8A). Postepigastric scutum long, strongly sclerotized, venter fully occupied, posteriorly directed lateral apodemes (lap) long (Figs 7B, 8B). *Legs*: yellowish white. Genitalia. *Epigastric region*: sperm pore situated at level of posterior spiracles (Fig. 8B). *Palp*: White, not strongly sclerotized (Fig. 9A); cymbium slightly rectangular in dorsal view; bulb stout; conical bulge slightly flat (Fig. 9A, B); embolus (e) basally narrowed, very long, twisted and with wavy tip; conductor (c) medially triangular and with wide tip (Fig. 9B).

***Female (paratype)*.** Total length: 1.46; carapace length: 0.61; carapace width: 0.43; abdomen length: 0.85; abdomen width: 0.48. Colouration: same as in male. Cephalothorax. *Carapace*: Same as in male. *Mouthparts*: chelicerae distally without pointed tooth-like projection (tlp). Abdomen: Dorsal scutum not covering full length of the abdomen, soft tissue visible in dorsal view (Fig. 7C); epigastric scutum not fused to postepigastric scutum; postepigastric scutum not fully covering the venter, and with short posteriorly directed lateral apodemes (lap) (Fig. 8D). *Legs*: yellowish white. Genitalia. Dorsal view (Fig. 9C): Receptaculum (re) small, slightly rectangular; median part with papillae; laterally framed by rectangular sclerites (apodemes, lap) with wide and slightly bumpy tip, apodemes sloping upward; rectangular appendix lying dorsally of receptaculum about as long as receptaculum; a transverse sclerite (tsc) lies anteriorly to the receptaculum and bears medially a nail-like structure (na).

##### Natural History.

Specimens were collected in a secondary degraded lowland rainforest only.

##### Distribution.

Known only from the type locality, Bukit Duabelas National Park, Sumatra.

## Supplementary Material

XML Treatment for Aposphragisma

XML Treatment for Aposphragisma globosum

XML Treatment for Aposphragisma jambi

XML Treatment for Aposphragisma sumatra
